# Inflammasomes Are Influenced by Epigenetic and Autophagy Mechanisms in Colorectal Cancer Signaling

**DOI:** 10.3390/ijms25116167

**Published:** 2024-06-03

**Authors:** Györgyi Műzes, Ferenc Sipos

**Affiliations:** Immunology Division, Department of Internal Medicine and Hematology, Semmelweis University, 1088 Budapest, Hungary

**Keywords:** inflammasome, epigenetics, autophagy, regulation, colitis, colorectal cancer, tumor microenvironment, therapeutic target

## Abstract

Inflammasomes contribute to colorectal cancer signaling by primarily inducing inflammation in the surrounding tumor microenvironment. Its role in inflammation is receiving increasing attention, as inflammation has a protumor effect in addition to inducing tissue damage. The inflammasome’s function is complex and controlled by several layers of regulation. Epigenetic processes impact the functioning or manifestation of genes that are involved in the control of inflammasomes or the subsequent signaling cascades. Researchers have intensively studied the significance of epigenetic mechanisms in regulation, as they encompass several potential therapeutic targets. The regulatory interactions between the inflammasome and autophagy are intricate, exhibiting both advantageous and harmful consequences. The regulatory aspects between the two entities also encompass several therapeutic targets. The relationship between the activation of the inflammasome, autophagy, and epigenetic alterations in CRC is complex and involves several interrelated pathways. This article provides a brief summary of the newest studies on how epigenetics and autophagy control the inflammasome, with a special focus on their role in colorectal cancer. Based on the latest findings, we also provide an overview of the latest therapeutic ideas for this complex network.

## 1. Introduction

The role of the inflammasome in inflammation is receiving increasing attention, as inflammation has a protumor effect in addition to inducing tissue damage [1]. The inflammasome’s function is complex and regulated on many levels. The role of epigenetic processes in its regulation is significant and has been extensively investigated, as it involves many potential therapeutic targets. The regulatory processes between the inflammasome and autophagy are also highly complex, with both beneficial and detrimental effects. The regulatory elements between the two also involve a number of therapeutic targets.

Inflammasomes exert profound effects on colonic carcinogenesis by regulating tumor-promoting inflammation within the tumor microenvironment (TME) [2]. Given the importance of epigenetics and the autophagy apparatus in regulating inflammasomes, their interconnected functions have the potential to modify cancer signaling [3].

Colorectal cancer (CRC) is a prevalent kind of cancer that affects both males and females globally. As per the American Cancer Society [4], the likelihood of acquiring CRC throughout a lifetime is expected to be 4.3% for men and 4.0% for women. In 2020, CRC accounted for 10% of new cancer cases and 9.4% of all cancer-related deaths [5]. The prevalence of colitis-associated cancer (CAC) in people with inflammatory bowel disease (IBD) is believed to rise steadily in correlation with the length of time the condition is present. The cumulative risks of CAC were 1%, 2%, and 5% after disease durations of 10 years, 20 years, and more than 20 years, respectively [6,7].

Mutations that modify genetic material are crucial determinants of cancer progression. They can be categorized as either somatic, acquired intermittently throughout one’s lifespan, or inherited, resulting in a predisposition to developing colorectal cancer [4,5]. Primary tumor localization is one of the most significant determinants of mutation patterns within colorectal cancer cells. Right-sided malignancies are associated with CIMP, MSI, and BRAF and KRAS gene mutations that occur more frequently [4,5]. There is an association between left-sided malignancies and a higher incidence of chromosomal instability. The Wnt/APC/β-catenin signaling pathway is the most crucial pathway implicated in CRC carcinogenesis. Indeed, it has been documented that at least one regulator of Wnt signaling is altered in 92% of sporadic CRC cases, with APC being the most widely recognized one [4,5]. CAC is a unique subtype of CRC, distinguished by its etiology. In IBD, chronic intestinal inflammation serves as the source of CAC. Because they help immune cells release oxidative enzymes and create reactive oxygen and nitrogen species, pro-inflammatory cytokines cause changes in epithelial cells during the course of IBD. These modifications impact the functionality of intestinal epithelial cells’ life cycles and influence their additional characteristics, such as migration, ultimately culminating in the development of cancer and the spread of metastases. Distinct mutation patterns were observed in CAC compared with sporadic CRC. One fundamental difference between CAC and sporadic CRC is that p53 mutations occur earlier in CAC, whereas APC and KRAS mutations occur later and less frequently [4,6].

Although inflammation is the primary driver of CRC development, its function varies depending on the CRC subtype. Inflammation initially propels CAC, but in sporadic CRC, the systemic inflammatory response acts more as an effect than a cause of cancer progression. The correlation between the activation of inflammasomes, autophagy, and epigenetic changes in CRC is intricate and encompasses several interconnected pathways. This article aims to present a concise overview of how epigenetics and autophagy regulate the inflammasome, specifically concerning colorectal cancer, based on the latest research findings.

## 2. Structure and Types of Inflammasomes

Since numerous excellent reviews of inflammasomes’ structure and function have been published (e.g, [8,9]), we will only reiterate the most essential details. In general, inflammasome protein complexes comprise the following components: a sensor, an adaptor (caspase-1), and an effector (ASC) [1]. The induction of pyroptotic cell death and the release of pro-inflammatory cytokines (e.g., IL1β and IL18) are the functional results of inflammasome activation. Pyrin-type, NLR, and ALR inflammasomes are the three classifications of inflammasomes, based on the sensor protein. The structural characteristics of the NLR family members include the central NBD and the C-terminal LRR domain. The variable is the third N-terminal domain. Two subgroups of NLRs are possible, according to the configuration of the third domain. The NLRP group comprises members including NLRP1 and NLRP3, both of which possess a PYD sequence at their N termini. The second group is the NLRC family, which comprises NLRC4 and other members with N-termini that contain CARDs [8,9].

**Figure 1:**
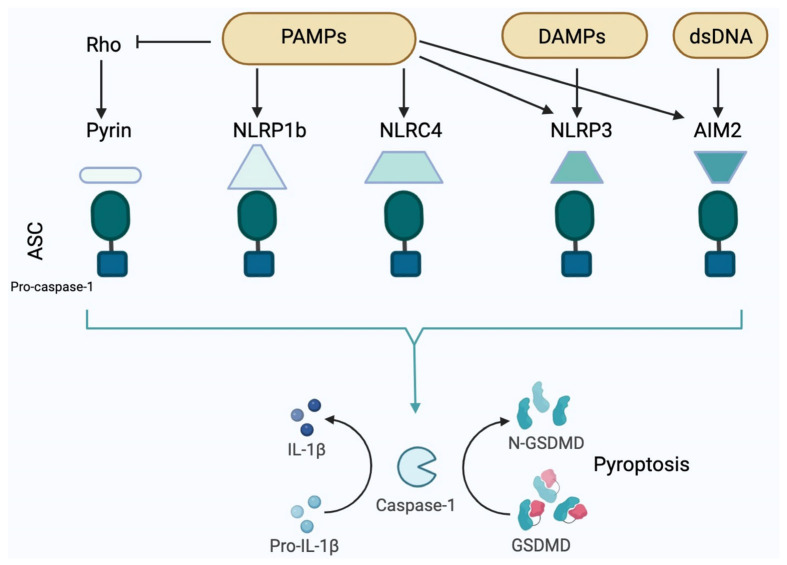
Classification and mode of action of various inflammasomes. There are different PAMPs that can turn on the NLRP3, NLRP1, NLRC4, AIM2, and pyrin inflammasomes. NLRP3 is also capable of detecting uric acid crystals, mtDNA, ROS, ATP, and mineral particulates (e.g., asbestos or silica), among others. AIM2 interacts structurally with particular ligands, including dsDNA. Rho modification triggers the activation of pyrins in response to toxins produced by bacteria. Upon activation, the sensors recruit and stimulate caspase-1. If an inflammasome has a CARD domain, then it can interact directly with pro-caspase-1. If it only has a pyrin domain, then it does so through the adaptor protein ASC. IL1β and IL18 are produced when pro-IL1β and pro-IL18 are cleaved and activated by caspase-1, respectively. Additionally, caspase-1 mediates the cleavage of gasdermin D to induce pyroptosis. The figure was partially created with https://www.biorender.com (accessed on 28 April 2024).

Activation of the NLRP3 inflammasome occurs through two signals, namely activation of the TLR/TNFR-mediated NFκB pathway or assembly of the inflammasome complex triggered by particulate matter, such as lysosomal destabilization or cathepsin B release, mitochondrial ROS generation, intracellular calcium influx, or potassium efflux [8,9]. The active NLRP3 inflammasome facilitates the development of IL1β and IL18 and triggers pyroptotic cell death, which refers to the osmotic lysis of cells. The assembly of the AIM2 inflammasome is triggered by the detection of DNA in the cytoplasm, resulting in pyroptosis and the maturation of IL1β and IL18 [8,9] (Figure 1).

## 3. Inflammasomes in CRC

There is substantial evidence to imply that the NLRP3 inflammasome plays a role in determining the prognosis, treatment response, and progression of tumors [10,11]. Conversely, empirical evidence suggests that NLRP3 plays a pivotal role in thwarting tumorigenesis via its ability to regulate host immunity [12]. Therefore, it is possible to characterize the NLRP3 inflammasome as a double-edged weapon for cancer, in that it may either inhibit the development of specific forms of cancer or, conversely, promote their occurrence. An increasing body of evidence indicates that dysregulation of the inflammasome pathway may play a role in the development and progression of CRC as well. Through their involvement in chronic inflammation and the production of pro-inflammatory cytokines, induction of tumor-promoting factors, modulation of the gastrointestinal microbiota, and regulation of immune responses, inflammasomes contribute to the development of CRC. Gaining insight into the molecular processes that govern the activation of the inflammasome in CRC could potentially pave the way for the creation of innovative therapeutic approaches aimed at preventing and treating this ailment.

### 3.1. Effect of Inflammasomes on Tumor-Promoting Inflammation

Experiments on genetically modified animals, analyses of colon adenocarcinoma cell lines, and human CRC and CAC samples have unveiled the roles of various inflammasome components in relation to colon inflammation and cancer [13]. It was noticed that rodents lacking NLRP3, ASC, Casp-1, or IL18 displayed diminished levels of IL18, as well as impaired activation and production of IFNγ and STAT1. These variables may contribute to an increased susceptibility to DSS-induced colitis and the progression of CAC [14]. A notable finding was that rodents lacking NLRP6 and ASC displayed an elevation in Csnk1ε, a protein that stabilizes β-catenin. Additionally, the expression of IL18 decreased, while the functionality of SMARRC1, IL6, and CCL5 increased. The combination of these factors may give rise to mechanisms that promote inflammation and tumor growth [15,16,17]. An increased pro-inflammatory cytokine profile including TNFα, IL6, and IL1β was identified in rodents lacking NLRP6 [18]. A severe enhancement in acute and recurrent colitis as well as the progression of CAC was observed in rodents lacking NLRP3, Pycard, and Casp-1, which was partially induced by suppressed IL1 and IL18 [19]. In mice lacking NLRP3, NLRC4, and Aim2, the expression of not only IL18 but also FasL was reduced, resulting in the development of CAC metastases in the liver [20]. Increased NFκB and decreased IL18 expression also contributed to the progression of colitis and CAC tumorigenesis in Casp-1- and Casp-12-deficient animals [21,22]. Deficiencies in Casp-1 and NLRC4 were associated with increased proliferation and apoptosis of colonic epithelial cells [22]. In rodents, Aim2 deficiency increased the risk of colon tumorigenesis via dysregulated Wnt signaling, as well as increased DNA-PK and Akt activation [23,24,25]. In NAIP1-6-deficient mice, the elevated STAT3 activity and reduced p53 function led to a higher susceptibility to CAC [26]. These results show that disruption of inflammasome function has a mostly protumor effect.

However, activation of the inflammasome can also have a protumor effect. NLRP3 inflammasome activation is associated with the EMT process and has been identified as a factor in the progression of sporadic CRC [27]. Survival in patients with CRC positively correlates with NLRP3 expression. This indicates that NLRP3 targeting may represent a promising form of treatment for CRC [28]. The correlation between tumor progression and epithelial expression of NLRP6 and IL18 establishes NLRP6 and IL18 expression levels in sporadic CRC cells as robust predictors of patient outcomes [29]. The therapeutic benefits of restoring normal NLRP6 expression in sporadic CRC cells may surpass those of disease progression prevention.

### 3.2. Inflammasomes Modulate the Tumorous Microenvironment and Tumor-Associated Macrophages

Aberrations in inflammasome function in the TME and through tumor-associated macrophages (TAMs) also influence colon carcinogenesis.

Despite the transcriptional heterogeneity of TAMs, little has been known about their spatial distribution and cell interactions which determine their tissue functions [30]. Recently, five discrete populations of human macrophages were identified spatially within healthy and malignant colon tissues. Spatial segregation of macrophage populations within microenvironmental niches containing conserved cellular compositions that are replicated in both healthy and diseased tissue was observed. It was discovered that IL4I1+ macrophages phagocytose deceased cells in regions with high cell turnover and predict a favorable prognosis for sporadic colon cancer. SPP1+ macrophages, on the other hand, are overrepresented in necrotic and hypoxic tumor regions and are indicative of a poorer prognosis in colon cancer. FOLR2+ macrophages are present in plasma cell compartments as a subset. Tumors contain NLRP3+ macrophages which co-localize with neutrophils and activate an inflammasome [31].

Prolonged inflammation is a major contributor to CAC. IL1β and other inflammatory cytokines have been implicated in the promotion of CAC [32]. ZNF70 is an integral component of numerous biological processes. ZNF70 was found to modulate the secretion of IL1β by macrophages, which promotes the proliferation of the HCT116 colon cancer cell line. Clinical CAC samples showed significantly higher ZNF70 expression compared with adjacent normal tissues. ZNF70 also stimulated macrophage IL1β secretion and HCT116 cell proliferation, predominantly via STAT3 activation. Moreover, ZNF70 activated the NLRP3 inflammasome in THP-1 cells stimulated with LPS/ATP, leading to robust IL1β secretion. The interaction between the ZnF domain of ZNF70 and NLRP3, which inhibits the K48-linked ubiquitination of NLRP3, underlies this phenomenon. Using the TCGA database, the link between ZNF70 expression and overall survival or IL1β expression in CAC was further confirmed [33].

Colonic epithelial cells effectively deliver self-derived and pathogen antigens to intraepithelial T cells in response to IFNγ detection [34]. Antigen presentation by epithelial cells to associated intraepithelial T cells confers extracellular ATPase expression. This inhibits the accumulation of extracellular ATP and, by extension, activation of the NLRP3 inflammasome in tissue macrophages [34]. On the other hand, the pathogenic transformation of CD4+ T cells into GM-CSF-producing T cells in vivo, triggered by antigen presentation by tissue macrophages and the production of IL1α and IL1β associated with the inflammasome, facilitates the promotion of colitis and CAC [34].

The NLRP3 inflammasome, PARP-1, and autophagy all play different roles in the TME and other tissues outside of the gut. These molecular parts interact with each other in complex ways [35]. Plasma LPS concentrations were considerably increased in AOM/DSS mice when compared with the control group. Moreover, activated macrophages and neutrophils secreted nitrites, which could potentially pose a cancer risk to patients with UC [35]. AOM/DSS treatment significantly increased the nitrite concentration in colon tissue compared with the control group. Nitrite inhibits the proliferation of cancer cells when present in trace amounts. However, its presence in high concentrations has been found to promote the progression of advanced-stage CACs [35].

### 3.3. Interaction of the Inflammasome with PAMPs and DAMPs Affects Immune Responses

Pretreatment with *E. faecalis* or NLRP3 siRNA has been shown to inhibit NLRP3 inflammasome activation in macrophages and slow the progression of colorectal tumors associated with colitis in mice [36]. Furthermore, the application of atractylenolide I significantly reduced NLRP3 inflammasome activation and cell survival in human colon cells. Additionally, it inhibited the development of colon tumors in the AOM/DSS mouse model [37]. NLRP3 expression was also found to be upregulated in colon adenocarcinoma tissues obtained from sporadic CRC patients, according to clinical findings [38]. Furthermore, a positive correlation has been observed between elevated levels of NLRP3 expression and malignant tumors, distant metastasis, vascular invasion, and lymph node positivity [38].

The gut mucosal DAMPs S100A8 and S100A9 have recently come to be recognized as pivotal agents in the pathogenesis of colonic inflammation. They interact with their PRRs, notably TLR4 and RAGE [39]. In murine colitis and CAC models, elevated serum concentrations of S100A8 and S100A9 were detected, in addition to notable enhancements in their interaction with PRRs, especially TLR4, and RAGE [40]. A recently developed CT peptide (rCT-S100A8/A9) was conjugated with a dual PRR-inhibiting peptide system comprising motifs derived from S100A8 and S100A9 that inhibit TLR4 and RAGE [41]. This peptide system was designed to facilitate colon-specific delivery. The administration of rCT-S100A8/A9 via injection into mouse models of acute and chronic DSS-colitis resulted in a notable enhancement of survival rates and amelioration of pathological lesions in the colon. In the AOM/DSS-induced CAC murine model, rCT-S100A8/A9 injection substantially ameliorated histological and clinical disease activities and decreased the tumor burden in the distal colon. Injection of rCT-S100A8/A9 into mice harboring oxaliplatin-resistant colorectal cancer xenografts substantially impeded tumor growth, as measured by decreased expression of EMT-associated markers in tumor tissues. To explore the potential involvement of S100A8 (or S100A9) in activation of the NLRP3 inflammasome, an investigation was conducted to determine whether its interaction with TLR4 or RAGE contributed to NLRP3 inflammasome activation. An NLRP3 inflammasome inducer (LPS/ATP) was discovered to increase extracellular S100 secretion and significantly enhance the interaction of S100 with TLR4 and RAGE in both BMDMs and THP-1 cells when S100A8 (or S100A9) was expressed. Additionally, these interactions were discovered to be effectively inhibited by A8 or A9 peptides. By demonstrating that targeting the S100-NLRP3-PRR axis reduces colonic inflammation, these results underscore the potential therapeutic utility of this axis in the treatment of colitis and CAC [41].

The energy homeostasis-associated (ENHO) gene encodes adropin, which is abundant in several tissues. Disorders of the central nervous system, metabolism, inflammation, and immunity are all linked to abnormal adropin levels [42]. It has been shown that tumor nest cells in advanced-stage sporadic CRC express less adropin [43]. The correlation between adropin expression by carcinoma cells and macrophage infiltration in the TME was negative. Conversely, adropin expression was upregulated by TAMs, which exhibited a positive correlation with both tumor invasion and metastasis. Transfection of MC38 colon cancer cells with the ENHO gene inhibited tumor growth in vivo, concomitant with an increase in M1 macrophages. Ex vivo treatment of macrophages with low-dose adropin increased mitochondrial ROS, specifically for inflammasome activation. In addition, ENHO^−/−^ mice exhibited a reduced number of M1 macrophages in vivo, and ex vivo, ENHO^−/−^ macrophages were incapable of being induced to form the M1 subset. Adropin at moderate doses increased glucose utilization, whereas adropin at high doses increased CPT1α expression in macrophages. Thus, differences in the concentration of adropin in carcinoma cells or macrophages within tumor tissues play distinct roles in the progression of sporadic CRC. Adropin at a low dose induces the antitumor activity of macrophages, whereas adropin at a high dose promotes the proliferation of tumor cells. Adropin levels can impede the progression of sporadic CRCs at various stages [43].

The γδT cells are the primary secretors of IL17A in the sporadic CRC TME [44]. IL17A regulates the TME in a variety of ways. IL17A may promote intracellular ROS accumulation and induce mitochondrial dysfunction and pyroptosis via the ROS/NLRP3/caspase-4/GSDMD pathway [45]. Furthermore, IL17A has the ability to incite CD8+ T cells to invade tumors and stimulate the release of inflammatory factors, including IL1β, IL18, and immune antigens [45]. Targeting inflammation via the IL17A-pyroptosis pathway may also have potential anticancer therapeutic efficacy.

### 3.4. The Connection between Diet, the Microbiome, and the Inflammasome

Induced by a high-fat diet (HFD), treatment with exogenous butyrate reverses the M1 polarization of proinflammatory macrophages, exacerbates intestinal inflammation, and accelerates tumor growth [46]. Activation of the NLRP3/caspase-1 pathway in the colon by HFD is also substantially inhibited. To determine the level of M1 polarization and expression of the NLRP3/caspase-1 pathway, LPS combined with butyrate was utilized to treat macrophages in vitro. The results were consistent with those obtained from in vivo experiments [46]. HFD promotes the development of colitis-associated tumors by disrupting the microbial community in the intestine and obstructing the metabolism of butyrate, which alters the polarization of macrophages. A viable new treatment strategy for CAC, exogenous butyrate, has promising clinical application prospects.

Inhibition of NLRP3 by the small molecule inhibitor MCC950 reduces DSS-colitic mouse disease activity by a significant amount [47]. Also, the overactivated NLRP3/ASC/caspase-1/IL1β signaling pathway in the colon was impeded by NLRP3 inhibition. In addition, the gut microbiome was modified by MCC950, which led to an increase in the abundance of Firmicutes, a decrease in the abundance of Bacteroidetes, and an increase in the ratio of Firmicutes to Bacteroidetes [47]. These results suggest that inhibiting the NLRP3 inflammasome could potentially regulate the abundance of intestinal flora. Based on correlation analysis, NLRP3 may exert its regulatory function on oxidation indicators through modulation of the composition of the gastrointestinal microbiota, particularly the phylum Bacteroidota, genus *Lactobacillus*, and species *Lactobacillus reuteri* [47].

**Figure 2:**
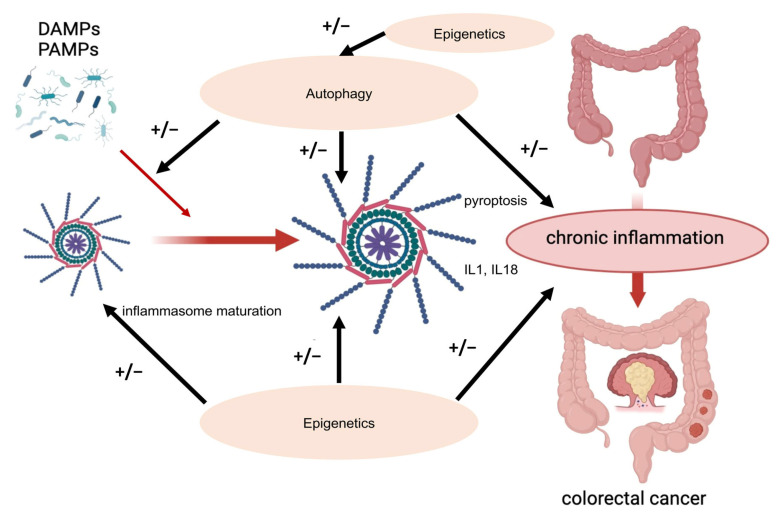
The NLRP3 inflammasome pathway in CRC. PAMPs and DAMPs have the ability to trigger the activation of NLRP3 inflammasomes. The activation of NFκB and subsequent overexpression of NLRP3, triggered by microbial components or endogenous cytokines, initiates the generation of pro-IL1β and pro-IL18, leading to chronic inflammation in CRC. The inflammasome is under multi-level regulation. This regulatory network includes epigenetics and autophagy machinery. The figure was partially created using https://www.biorender.com (accessed on 28 April 2024).

All these data clearly show that the inflammasome can influence colorectal cancer development, progression, and metastasis through several mechanisms. It follows that such an important mechanism must be multi-regulated to preserve physiological conditions. At the same time, possible defects or derailments in multifaceted regulation can affect colorectal tumor formation and progression (Figure 2).

## 4. Epigenetic Regulation of Inflammasomes in CRC

In cancers, the inflammasome is regulated epigenetically through alterations to the structure of DNA and any associated proteins. These modifications can affect gene expression without modifying the underlying DNA sequence. Epigenetic modifications significantly contribute to dysregulation of the inflammasome pathway.

### 4.1. Methylation of Inflammasome-Associated Genes

In CRC and CAC, aberrant DNA methylation patterns have been observed in genes associated with inflammasome activation, such as NLRP3 and ASC, contributing to inflammasome dysfunction.

In a pan-cancer analysis of NLRP3 inflammasome methylation, it was found that in sporadic CRC, seven NLRP3 inflammasome-related genes (i.e., CARD8, ATAT1, CD36, NLRC3, NLRP3, PSTPIP1, and TXNIP) showed altered DNA methylation statuses. Regarding NLRP3 inflammasome gene expressions, general downregulation was detected [48].

The binary TMS1/ASC protein (also called PYCARD) was recognized as an adaptor molecule of the inflammasome that facilitates the activation of caspase-1 and the cleavage and maturation of pro-IL1β and pro-IL18, and thus it helps with promoting inflammation [49,50]. TMS1/ASC is made up of two protein–protein interaction domains: a pyrin domain and a caspase recruitment domain. TMS1/ASC may indirectly induce chronic inflammation, angiogenesis, activation of the IL17 pathway, myeloid-derived suppressor cell differentiation, macrophage recruitment, invasion, and metastasis through the promotion of inflammation and, more specifically, the release of IL1 [51]. Proteins containing these domains play a critical role in the regulation of immune response pathways and apoptosis. Mutations occurring in several proteins containing PYD and CARD have been associated with autoinflammatory disorders and malignancy [52]. Using methylation-specific PCR, the methylation status of TMS1/ASC was determined in a variety of colorectal adenomas, primary sporadic CRCs, and normal colon samples [53]. Methylation analysis showed complete methylation of TMS1/ASC in 71% of the CRC cell lines. The same cell lines exhibited an absence of mRNA expression, as determined by RT-PCR. Treatment with the demethylating agent restored expression. Methylation was detected in 17% of primary sporadic CRC specimens but not in adenomas or the normal samples. According to multivariate analysis, the methylation status of TMS1/ASC was correlated with a series of clinicopathological variables. In agreement with hMLH1 methylation, TMS1/ASC methylation was more prevalent in lesions located on the right side. It appears that methylation of TMS1/ASC occurs during the late stages of colorectal carcinogenesis and is exclusive to invasive carcinomas [53].

Hypomethylation of NLRP3, NLRP12, and NLRC4 has been identified in patients with severe UC [54]. Hypomethylation of NRLP inflammasomes in response to inflammation may deliver anti-inflammatory signals with the potential to ameliorate severe colitis, impede additional damage, and prevent the initiation of CAC [55]. Additionally, in chronic UC, the hypomethylation of NLRP12 and NLRC4 may modulate the gut microbiota to prevent intestinal inflammation and subsequent intestinal injury [54].

The causal relationship and putative molecular mechanisms underlying sporadic CRC and atopic dermatitis were assessed in a recent study through the integration of Mendelian randomization and multiple transcriptome analyses [56]. A causal relationship was identified between AD and a reduced incidence of sporadic CRC. Furthermore, a shared gene signature between AD and CRC was identified in TET2, an epigenetic regulator. The expression of TET2 was observed to be decreased in both AD and CRC, with a correlation between downregulation and an unfavorable prognosis in CRC. It has been previously established that TET2 and the IL1β/NLRP3 inflammasome are molecularly linked [57]. There was a significant correlation, as determined by methylation analysis, between the downregulation of TET2, elevated methylation of cg09666717 and cg12306086, and poor prognosis in sporadic CRC [56].

The results presented here indicate that regulation of inflammasomes by DNA methylation plays a role in the development of CRC, but further targeted and detailed studies are needed to map the methylation statuses of inflammasome-associated genes in CRC.

### 4.2. Post-Translational Modifications Affecting the Tumorigenic Function of the Inflammasome

Histone proteins are susceptible to a range of post-translational modifications, which have an impact on both gene expression and chromatin structure [58]. These modifications include acetylation, methylation, phosphorylation, and ubiquitination. The expression of genes associated with inflammasomes can be modulated by altering the accessibility of these genes to transcription factors and RNA polymerase machinery via histone modifications at their promoter regions. Histone deacetylases, for instance, are capable of eliminating acetyl groups from histones, thereby inducing chromatin condensation and transcriptional repression. Deregulation of inflammasomes in colorectal cancer has been linked to dysregulation of histone modifications.

HDAC6 is implicated in the processes of inflammasome assembly, priming, and activation [59]. Primarily through deacetylation and activation of the p65 subunit of NFκB, which subsequently stimulates the transcription of genes encoding NLRP3, pro-IL1β, and pro-IL18, HDAC6 facilitates priming of the NLRP3 inflammasome [60]. Moreover, by activating PrxII, HDAC6 induces the activation of inflammasomes, thereby increasing the level of ROS [61]. The inhibition of HDAC6 increases p65 expression in the cytoplasm of macrophages but decreases p65 expression in their nuclei, thereby inhibiting NLRP3 transcription and pyroptosis [62]. On the contrary, inflammasome activation is negatively regulated by HDAC6 via its interaction with ubiquitinated NLRP3 [63].

Non-coding RNAs, such as microRNAs and long non-coding RNAs, can modulate the stability or translation of target mRNAs, thereby regulating gene expression. In CRC, different miRNAs and lncRNAs have been identified as regulators of inflammasome components.

In the transcriptionally active phase, miR-223 has the potential to modulate NLRP3 mRNA [64], and miR-223 can attach to a highly conserved region of the 3′UTR of NLRP3 mRNA and consequently inhibit protein translation, according to an in silico analysis [65]. While miR-223 has been associated with decreased NLRP3 expression and IL1β secretion in inflammatory circumstances, there is inconclusive evidence regarding its effects in sporadic CRC [66,67,68]. A recent study revealed that wild-type miR-223 may exert a more potent anticancer effect in sporadic CRC when compared with glibenclamid [69]. However, it is important to note that relying solely on miR-223-mediated NLRP3 suppression may not be adequate to prevent CRC metastasis.

Meanwhile, miR-203, in part by modulating the expression of NLRC4, may serve as a biomarker for monitoring the progression and survival outcomes of sporadic CRC [70].

Consensus molecular subtypes of CRC have been linked to lncRNAs, revealing a complex interaction between lncRNAs, inflammation, and immunology in the tumor stroma [71]. By acting as molecular reservoirs for miRNAs, certain lncRNAs may alleviate the repression of genes associated with inflammasomes. After constructing an lncRNA signature associated with pyroptosis (i.e., ELFN1-AS1, PCAT6, TNRC6C-AS1, and ZEB1-AS1), a novel prognostic indicator for colorectal cancer has been identified. Functional validation of this lncRNA signature was conducted with an independent cohort of sporadic CRC patients [72].

Recent research has brought attention to the significance of ubiquitination, which is a post-translational modification, in both the initiation and progression of colonic inflammation [73]. The deubiquitinating enzyme ovarian tumor deubiquitinase 6A (OTUD6A) controls both cell proliferation and tumorigenesis. In vivo, OTUD6A deficiency was observed to result in reduced severity of colitis induced by DSS or TNBS, as well as CAC induced by AOM/DSS. Additionally, bone marrow transplantation experiments demonstrated that the exacerbation of DSS-induced colitis was caused by OTUD6A in myeloid cells. The NLRP3 inflammasome’s NACHT domain was directly bonded to by OTUD6A, which cleaved selectively K48-linked polyubiquitin chains from NLRP3 at K430 and K689 in order to improve the protein’s stability. This process ultimately resulted in the upregulation of IL1β and inflammation [73].

## 5. Inflammasome and Autophagy Regulatory Crosstalk in CRC

Autophagy is a biological mechanism that enables cells to adapt to several types of stress, including insufficient nourishment, infection, and a lack of oxygen. This mechanism is crucial for maintaining cell homeostasis by supplying cells with nutrients during periods of stress and eliminating damaged mitochondria and mitochondrial DNA. Autophagy is linked to several physiological processes, including the elimination of germs from cells [74], the release of inflammatory cytokines [75], regulation of inflammation, antigen presentation [76], and the development of lymphocytes [77]. Dysfunction in autophagy can lead to heightened inflammation and an elevated susceptibility to cancer [78]. The cross-regulation between the inflammasome and autophagy pathways in CRC involves complex interactions that influence various aspects of tumorigenesis, including inflammation, cell survival, and immune responses. Autophagy dysregulation can result in heightened inflammasome activation as a consequence of the buildup of impaired mitochondria and other signals that activate inflammasomes.

### 5.1. Impact of Autophagy and Inflammasome Crosstalk on Inflammatory Cytokines

**Figure 3:**
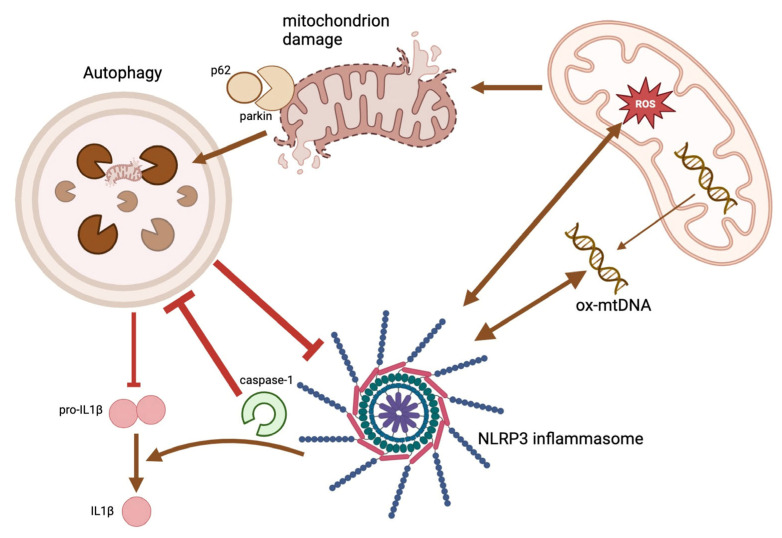
Inflammasomes and autophagy engage in cross-communication. By eliminating endogenous inflammasome activators, such as damaged mitochondria that generate reactive ROS, inflammasome components, and cytokines, autophagy can negatively regulate NLRP3 inflammasome activation. In addition to regulating the inflammatory response, autophagic machinery prevents the unusual secretion of IL1β. On the other hand, when the NLRP3 inflammasome activates, a number of distinct mechanisms govern the formation of autophagosomes. The maintenance of equilibrium between the necessary inflammatory response of the host’s defenses and the prevention of excessive inflammation require cross-communication between inflammasomes and autophagy. The figure was partly created with https://www.biorender.com (accessed on 28 April 2024).

The inflammasome and autophagy pathways have the ability to impact the generation and release of pro-inflammatory cytokines, including IL1β and IL18. Inflammasome activation results in the processing and release of IL1β and IL18. Autophagy can regulate the production of these cytokines by controlling the movement of vesicles containing pro-IL1β and pro-IL18 [79]. An imbalance in either route might disturb the equilibrium of pro-inflammatory cytokines, which can contribute to the advancement of CRC (Figure 3).

### 5.2. Effects of Autophagy and Inflammasomes on Tumor Cell Survival and Growth

Autophagy and inflammasome activation have the potential to impact the survival and growth of tumor cells in sporadic CRC and CAC. Autophagy enhances the survival of tumor cells by supplying nutrients and eliminating impaired organelles, therefore facilitating tumor proliferation [78]. On the other hand, the activation of inflammasomes can trigger pyroptotic cell death, which effectively restricts the growth of tumor cells [2]. Nevertheless, continuous activation of the inflammasome can potentially contribute to the advancement of colon cancer by encouraging long-term inflammation and establishing a microenvironment that supports tumor growth [2].

Regulating autophagy is critical for the immune system’s ability to detect and eliminate tumors as well as induce cell death in cancer cells. Autophagic cell death in cancer cells can stimulate ATP signaling through purinergic receptors, either autocrine or paracrine, leading to activation of the NLRP3 inflammasome and the production of IL1β [80]. Specifically, ATP produced by dying cells acts as a potent catalyst for pro-inflammatory reactions in macrophages located in the TME, hence enhancing the host’s immune responses against tumors [80,81].

The results of a recent meta-analysis of four large cohorts indicate that the polymorphisms in DAPK2 and ATG5 autophagy-related genes have an impact on the risk of developing sporadic CRC. This influence is partially due to their effect on IL1β production [82]. IRGM, a risk factor for Crohn’s disease, has been found to restrict the activation of the NLRP3 inflammasome by hindering its assembly and facilitating its selective autophagy [83].

Multiple studies have verified the role of ATG16L1 and IRGM variations in the susceptibility to Crohn’s disease and have provided novel insights into the significance of macroautophagy in regulating infection, inflammation, immunology, and cancer [84,85,86].

Overexpression of ATG16L2 has been shown to be associated with a favorable outcome in sporadic CRC [87]. Furthermore, ATG16L2 hinders activation of the NLRP3 inflammasome by facilitating the formation of the ATG5-12-16L1 complex and promoting autophagy [88]. This also highlights the role of autophagy in colitis and CAC.

### 5.3. Immune Responses Altered by Autophagy–Inflammasome Interaction

Both autophagy and inflammasome activation play important roles in modulating immune responses in CRC. Autophagy can regulate antigen presentation and modulate the function of immune cells, such as macrophages and dendritic cells, thereby influencing anti-tumor immunity [89]. Inflammasome activation can trigger the release of DAMPs and pro-inflammatory cytokines, which contribute to immune cell recruitment and activation [90]. Dysregulation of either pathway can affect immune surveillance and promote immune evasion by CRC cells.

NLRP3 and NLRC4 activation in macrophages is partly driven by mitochondrial damage and the release of mtDNA [91]. Selective autophagy controls the magnitude of this response by sequestering mitochondria and mtDNA [92]. Additionally, mitophagy and the NLRP3 inflammasome are involved in CRC progression, and their crosstalk influences cancer cell survival [93].

Optineurin is classified as an autophagy receptor [94], but its expression, regulation, and role in the context of immunity, particularly tumor immunity, remain unidentified. Recent findings indicate a constant decrease in optineurin transcripts and proteins in cancer epithelial cells, specifically in the TME, whereas immune cells remain unaffected [95]. Optineurin exerts a direct inhibitory effect on NLRP3 [96]. This indirectly triggers mitophagy, which in turn suppresses NLRP3 [97].

The anti-inflammatory activity of NFκB is achieved through the induction of delayed accumulation of p62/SQSTM1, a classic selective autophagy receptor [98]. External stimuli that activate NLRP3 induce a type of caspase-1/NLRP3-independent mitochondrial damage, which results in the release of direct activators of the NLRP3 inflammasome, such as mtDNA and mtROS. Parkin-dependent ubiquitin conjugation occurs on damaged mitochondria, and p62 specifically recognizes these particles, thereby initiating their mitophagic clearance [98]. Macrophage-specific p62 ablation promotes macrophage mortality by inducing an inordinate accumulation of damaged mitochondria and inflammation dependent on IL1β. Thus, the NFκB-p62-mitophagy pathway functions as an intrinsic regulatory loop within macrophages. However, it is also involved in CRC development [99].

FUNDC1, another selective autophagy (i.e., mitophagy) receptor, exerts an impact on mitophagy in several types of malignancies, including CRC [100], leading to a reduction in caspase-1 levels [101].

The Fanconi anemia DNA damage repair pathway has been discovered to have a significant impact on the genetic susceptibility to sporadic CRC. Recently, mutations in FANC genes, including FANCC, have been identified in CRC patients who did not have mutations in established CRC susceptibility genes. This discovery emphasizes the possible involvement of FANC genes as predisposition genes in sporadic CRC [102,103]. The interaction between FANCC and Parkin promotes mitophagy, a process which suppresses inflammasome activation [104].

### 5.4. Regulatory Interaction between Autophagy and Inflammasomes

Autophagy has the ability to govern an inflammasome, and vice versa; the inflammasome may also affect the autophagy process. In general, activation of the inflammasome promotes autophagy, which subsequently suppresses inflammasome activity to prevent excessive activation.

NLRC4 and NLRP4 have a role in the progression of sporadic CRC [105,106]. The NLRC4 inflammasome has been discovered to control the development of liver metastases in colon cancer [107]. NLR family members are involved in cooperation with Beclin1. In this interaction, the NLR NACHT domain facilitates contact between Beclin1 and the heterologous NACHT domain. Suppression of NLRC4 or NLRP4 enhances the autophagic process. However, only NLRP4 and not NLRC4 hinders the development of autophagosomes [108].

The caspase-1/IL18 axis has been observed to have increased functional activity in colon cancer cells [109,110]. The involvement of beclin-1 increases the process of mitophagy through caspase-1 activation [109,110]. This affects the activation of a Th1/Tc1 immune response induced by IL18Rα-expressing tumor-infiltrating lymphocytes. Based on these data, it has been found that by focusing on the caspase-1/IL-18 axis, the effectiveness of the body’s immune response against tumors can be enhanced in some subgroups of sporadic CRC [109,110].

The Ras division of the Ras superfamily classifies RalA and RalB, which are two small GTPases [111]. Ral oscillates between phases of inactivity, during which it is linked to GDP, and phases of activity, during which it is attached to GTP. Ral-specific guanine nucleotide exchange factors and GTPase-activating proteins regulate this cycle. Although RalA and RalB share significant sequence similarity and exhibit comparable structural and biochemical characteristics, their effects on cancer cells, such as CRC cells, have been found to be markedly divergent [112]. Macrophages activate autophagy in response to various inflammasome stimuli via RalB nucleotide exchange. The combined inflammasomes underwent ubiquitination and attracted the autophagic adaptor p62, facilitating their transport to autophagosomes [113].

Autophagy can also influence inflammasome function in CRC at several points. In theory, epigenetic and autophagy regulation of inflammasome function can affect each other in a complicated way. This is made even more complicated by the fact that epigenetic changes can have an indirect effect on autophagy regulation of inflammasome function. DNA methylation, histone methylation, histone acetylation, miRNAs, and post-translational modification (e.g., acetylation, ubiquitination, or phosphorylation) may influence the expression of autophagy-associated genes. A detailed and exhaustive summary of this interplay was given by Shu et al. [114]. However, this multilevel regulatory system has led to the identification of a number of completely new antitumor therapeutic targets, which are of significance in daily practice.

## 6. Novel Therapeutical Aspects of Epigenetic- and Autophagy-Related Inflammasome Regulation in CRC

### 6.1. Therapeutic Agents with Anti-Inflammatory and Antitumor Properties

Irinotecan, also known as CPT-11, is a chemotherapeutic medication used for the treatment of CRC. Nevertheless, gastrointestinal toxicities, such as diarrhea and intestinal inflammation, significantly limited its use in clinical settings. *Aucklandia lappa* Decne. roots are utilized in traditional Chinese medicine for alleviating gastrointestinal problems. One of the primary active components of these roots is dehydrocostus lactone (DHL) [115]. Through investigation of the defensive properties of DHL against CPT-11-induced intestinal mucositis and its underlying processes, it was discovered that DHL effectively reduced the intestinal damage caused by CPT-11. Histological examination indicated that DHL effectively avoided damage to the intestinal epithelium and enhanced the function of the intestinal barrier in mice stimulated with CPT-11. In addition, DHL demonstrated its ability to reduce inflammation in CPT-11-induced intestinal mucositis by suppressing development of the TLR4/MD2 complex and subsequently modulating the NF-κB/NLRP3 signaling pathway. DHL has the potential to be used as a new approach for combining medicine with irinotecan [116].

Fisetin, also known as 3,7,3′,4′-tetrahydroxy flavone, is a kind of flavonol that may be found in a wide range of plants, fruits, and vegetables [117]. Fisetin has been documented to possess a variety of pharmacological characteristics, such as anticancer, antioxidative, and anti-inflammatory effects [118]. Recent observations have shown that fisetin hinders the creation and activation of the NLRP3 inflammasome by obstructing the TLR4/MD2 signaling pathway. Furthermore, it has been shown that p62-mediated mitophagy plays a vital role in suppression of the NLRP3 inflammasome by fisetin [119].

*Cnidium monnieri* (L.) Cuss contains the bioactive compound osthole. Studies have demonstrated several physiological effects of osthole, including its anti-inflammatory properties [120]. Osthole efficiently inhibits the proliferation of colorectal cancers and mitigates intestinal damage generated by AOM/DSS [121]. Osthole enhances the functionality of goblet cells and inhibits the expression of Claudin1 and Axin1, which are disrupted by AOM/DSS. Furthermore, osthole has cytotoxic effects, particularly on colon cancer cells, while having no impact on normal colon cells. Osthole inhibits the ASC/caspase-1/IL-1β inflammasome pathway and disrupts mitochondrial function by regulating redox and calcium balance. Furthermore, it hinders both oxidative phosphorylation and glycolysis, resulting in ATP synthesis inhibition. Additionally, studies have demonstrated that the combination of chloroquine and osthole disrupts the autophagy flux, leading to the programmed cell death of HCT116 and HT29 colon cancer cells. Furthermore, studies have clarified that the osthole-controlled functional role of tiRNAHisGTG directly influences the cellular destiny of colon cancer cells [121]. These findings indicate that osthole has the capacity to control the advancement of CRC by controlling the signal transmission of autophagy-inflammasome-mediated processes.

### 6.2. Therapeutical Agents Affecting Tumor Cell Kinetics

Proliferation, migration, and invasion of colon cancer cells were all substantially inhibited by cannabidiol (CBD) in a manner that was dependent on either the dose or time [122]. CBD has the potential to impede EMT via the downregulation of mesenchymal markers like N-cadherin, snail, vimentin, and HIF-1α and the upregulation of epithelial markers like E-cadherin. CBD could increase the expression of Axin1, inhibit the expression of β-catenin target genes, including APC and CK1, and suppress activation of the Wnt/β-catenin signaling pathway [122]. Studies have shown that dietary CBD effectively alleviates the symptoms of intestinal colitis, inhibits inflammation, and safeguards barrier function. The induction of PKA/AMPK signaling correlated with the observed outcomes. Furthermore, CBD supplementation successfully inhibited NLRP3 and MLCK signaling, facilitating the restoration of intestinal epithelium damaged by DSS [123]. CBD supplementation also suppressed NLRP3 inflammasome activation and related pro-inflammatory marker secretion [123].

PAMPs and DAMPs are NLPR3 inflammasome pathway triggers. Particular chemokines and receptor complexes play a role in this by promoting cellular proliferation and metastasis. Bacterial enzymes assault the colon mucosa in a synergistic manner, resulting in lysosomal discharge and cellular integrity degradation. These aforementioned factors additionally activate NLRs and TLRs, which facilitate angiogenesis and provide nutrients to cancer cells. Monoterpenes such as myrtenal and citronellol have become increasingly significant in recent years, finding extensive application in the treatment of various ailments, including cancer, neurodegeneration, and metabolic disorders. The therapeutic properties of monoterpenes, which inhibit inflammasomes, autophagy, and other signaling pathways [124,125], underpin their anticancer properties. Combination therapies are becoming increasingly significant due to their ability to target each variant of cellular stress. Positive results from clinical trials involving compounds in the monoterpene family may pave the way for the development of an effective form of anticancer drug therapy.

Arctigenin, the primary bioactive compound found in *Fructus arctii*, has been documented to hinder the proliferation of several types of cancer, possibly via inducing autophagy, and to relieve symptoms of colitis [126]. Orally administered arctigenin halted the advancement of colitis and provided protection against the development of CAC in AOM/DSS mice [127]. Arctigenin reduced activation of the NLRP3 inflammasome and the metabolism of fatty acid oxidation (FAO) in macrophages, as measured by untargeted metabolomics. Arctigenin suppressed the production of carnitine palmitoyltransferase 1 (CPT1), decreased the acetylation of α-tubulin, and disturbed formation of the NLRP3 complex, leading to inactivation of the NLRP3 inflammasome. Inhibition of the effect of arctigenin on NLRP3 inflammasome assembly was found when the CPT1-FAO-acetyl-coenzyme A-acetylated α-tubulin pathway was downregulated. It was also demonstrated that arctigenin can hinder activation of the NLRP3 inflammasome and enhance CAC in mice. Collectively, these findings demonstrate that the suppression of NLRP3 inflammasome formation in macrophages caused by FAO downregulation plays a role in the preventive impact of arctigenin against CAC [127]. The findings emphasize the possible efficacy of arctigenin in mitigating the risk of CAC in individuals with colitis.

### 6.3. Modulation of the Gut Microbiota and Related Immune Response

Intestinal cell inflammation and innate immune system disruption can result from dysbiosis. These effects include oxidative stress, redox signaling, electrophilic stress, and inflammation. In addition to promoting immune responses to the gut microbiota, the NLRP3 inflammasome regulates intestinal epithelial integrity. In a recent study, the therapeutic potential of 29 phytocompounds (e.g., artemisitene, morroniside, protopine, ferulic acid, quercetin, picroside II, and hydroxytyrosol) and 13 medicinal plants (e.g., *Litsea cubeba*, *Artemisia anomala*, *Piper nigrum*, *Morus macroura*, and *Agrimonia pilosa*) was assessed for in vitro and in vivo models of IBD [128]. The study specifically examined the impact of these compounds on the NLRP3 inflammasome. The interventions yielded the following outcomes: decreased levels of IL1β, TNFα, IL6, INFγ, and caspase-1; increased expression of antioxidant enzymes IL4 and IL10; and modulation of autophagy and the gut microbiota [128,129,130,131,132,133,134,135,136,137,138,139]. Potentially advantageous in the treatment of IBD and CAC, these effects may result in minimal or no adverse effects comparable to those of synthetic anti-inflammatory and immunomodulatory medications.

Observations show that CD82 inhibits NLRP3 inflammasome activation both in vitro and in vivo, and in rodents, CD82 deficiency reduces the severity of colitis [140]. CD82 is also involved in CRC pathogenesis [141]. Two BRCC3 and NLRP3 binding partners of CD82 have been identified. CD82 binding to these partners facilitated BRCC3-dependent K63-specific deubiquitination, thereby enhancing NLRP3 degradation. The CD82-specific bacteria *Bacteroides vulgatus* (*B. vulgatus*), which is present in the colon microbiota, regulates CD82 expression and facilitates activation of the NLRP3 inflammasome [140]. As a result, administering *B. vulgatus* to rodents with colitis improved survival by mediating CD82 expression and activating NLRP3. Suppression of CD82 decreased the pathogenesis of colitis by increasing NLRP3 inflammasome activation via BRCC3-dependent K63 deubiquitination [140]. Researchers have identified *B. vulgatus* as a contributor to CRC [142]. These results suggest *B. vulgatus* as an innovative therapeutic option for colitis and CRC.

### 6.4. Therapeutic Aspect of Neuro-Immunological Connections

Recent experimental evidence indicates that the interaction between neuro-immunological connections and autophagy might affect inflammasome function. Sacral nerve stimulation has been shown to improve DSS-induced colitis by increasing macrophage autophagy and suppressing the activation of NLRP3 inflammatory bodies [143]. This effect may be associated with activation of the cholinergic anti-inflammatory pathway. This approach has the potential to exhibit anti-cancer benefits by lowering inflammation.

## 7. Conclusions

The inflammasome may be aberrantly activated or inhibited during CRC development and progression and may exhibit cytoprotective, cell-damaging, oncogenic, or tumor-inhibitory functions. Although the multifactorial and heterogeneous nature of disease-relevant inflammasome function requires further elucidation, various in vitro and in vivo experimental studies have revealed a number of regulatory mechanisms that help to understand the deregulated function of the inflammasome. This review focused in part on epigenetic and posttranslational modifications, including classical DNA methylation, histone methylation and acetylation, microRNAs, lncRNAs, and ubiquitination. In addition, aspects of inflammasome regulation through autophagy were discussed in detail, taking into account the effects of the inflammasome on autophagy. We also mentioned ways in which the inflammasome regulates autophagy through epigenetic influences. In addition, we summarized new potential CRC therapeutic options that may involve manipulating the anti-inflammatory effect via autophagy or epigenetic regulation.

However, we still need to further explore and address a variety of limitations. Additional research is required to uncover the intercommunication between many epigenetic events that regulate inflammasomes in the TME of CRCs, including CAC. Furthermore, more research is required to investigate the influence of autophagy on the functions of inflammasomes in the context of CRC development and advancement. This research will help uncover the particular impacts of autophagy at different stages of CRC growth in an inflammatory setting. In order to possibly treat colorectal cancer, it is also important to understand the basic epigenetic signaling pathways that control the inhibition of autophagy-dependent inflammasomes. To fully understand how epigenetics and autophagy affect inflammasomes, we might be able to find new therapeutic targets that are useful for stopping the effects of inflammasome activation in the progression of CRC.

## Abbreviations

AD = atopic dermatitis; AIM2 = absent in melanoma 2; ALR = AIM2-like receptor; AMPK = adenosine monophosphate-activated protein kinase; AOM = azoxymethane; APC = adenomatous polyposis coli; ASC = apoptosis-associated speck-like protein containing a CARD (TMS, Pycard); ATAT = alpha-tubulin N-acetyltransferase; ATG = autophagy protein; ATP = adenosine triphosphate; BMDMs = bone marrow-derived macrophages; BRAF = B-type rapidly accelerated fibrosarcoma; BRCC3 = BRCA1/BRCA2-containing complex subunit 3; CAC = colitis-associated cancer; CARD = caspase recruitment domain; CBD = cannabidiol; CCL5 = chemokine (C-C motif) ligand 5; CIMP = CpG island methylator phenotype; CK1 = casein kinase 1; CPT-11 = irinotecan; CPT1 = carnitine palmitoyltransferase 1; CPT1 = carnitine palmitoyltransferase 1; CRC = colorectal cancer; Csnk1ε = casein kinase 1ε; DAMP = danger-associated molecular pattern; DAPK2 = death-associated protein kinase 2; DHL = dehydrocostus lactone; DNA-PK = DNA-dependent protein kinase; DNA = deoxyribonucleic acid; DSS = dextran sodium sulfate; ELFN1-AS1 = ELFN1 antisense RNA 1; EMT = epithelial-to-mesenchymal transition; ENHO = energy homeostasis-associated gene; FANC = Fanconi anemia complementation group; FAO = fatty acid oxidation; FasL = Fas-L (CD95L); FOLR2 = folate receptor beta; FUNDC1 = FUN14 domain-containing 1; GM-CSF = granulocyte-macrophage colony-stimulating factor; GSDMD = Gasdermin D; HDAC6 = histone deacetylase 6; HFD = high-fat diet; HIF = hypoxia-induced factor; hMLH1 = human mutL homolog 1; IBD = inflammatory bowel disease; IFN = interferon; IL = interleukin; IL4I1 = IL4-induced 1; IRGM = immunity-related GTPase family M protein; KRAS = Ki-ras2 Kirsten rat sarcoma viral oncogene homolog; lncRNA = long non-coding RNA; LPS = lipopolysaccharide; LRR = leucine-rich repeat; MD2 = myeloid differentiation factor 2; MLCK = myosin light chain kinase; mRNA = messenger RNA; MSI = microsatellite instability; mtDNA = mitochondrial DNA; NACHT = predicted nucleoside-triphosphatase (NTPase) domain; NAIP = NLR family, apoptosis inhibitory protein; NBD = nucleotide-binding domain; NFκB = nuclear factor-κB; NLR = NOD-like receptor; NLRC = NLR family CARD domain-containing protein; NLRP = NOD-like receptor protein; OTUD6A = ovarian tumor deubiquitinase 6A; p62/SQSTM1 = sequestosome 1; PARP-1 = poly (ADP-ribose) polymerase 1; PCAT6 = prostate cancer associated transcript 6; PKA = protein kinase A; PRR = pattern recognition receptor; PrxII = peroxiredoxin II; PSTPIP1 = proline-serine-threonine phosphatase-interacting protein 1; PYD = pyrin domain; RAGE = receptor for advanced glycation endproducts; Ral = small GTPase; RNA = ribonucleic acid; ROS = reactive oxygen species; S100 = S100 calcium-binding protein; siRNA = silencing RNA; SMARRC1 = a member of switch/sucrose nonfermentable chromatin remodeling complex; SPP1 = secreted phosphoprotein 1; STAT = signal transducer and activator of transcription; TAM = tumor-associated macrophage; TCGA = The Cancer Genome Atlas; TET2 = tet methylcytosine dioxygenase 2; TLR = toll-like receptor; TME = tumor microenvironment; TNBS = 2,4,6-trinitrobenzene sulfonic acid; TNF = tumor necrosis factor; TNFR = tumor necrosis factor receptor; TNRC6C-AS1 = TNRC6C antisense RNA 1; TXNIP = thioredoxin-interacting protein; UC = ulcerative colitis; Wnt = wingless/integrated; ZEB1-AS1 = ZEB1 antisense RNA 1; ZNF70 = zinc finger protein 70.
